
JOURNAL INFORMATION
==============================
NLM Title Abbreviation: Tex Med J (Austin)
NLM Journal ID: 21130590R

Texas Medical Journal

ISSN: 0892-8495
Publisher: Biomedical Journal Digitization Project

ARTICLE INFORMATION
==============================
PMCID: PMC9642202
PMID: 36956647
Article version: 1
Subjects: Abstracts and Selections

A Real Rip Van Winkle

Print publication date: 1911 Sep
Volume: 27
Issue: 3
First page: 113
Last page: 113
License: This work is free of known copyright restrictions. For more information, please see PMC Back Issue Digitization.
License URL: https://creativecommons.org/publicdomain/mark/1.0/

==============================

THE CHAS. H. PHILLIPS CHEMICAL CO.

NEW YORK and LONDON

PHILLIPS' MILK OF MAGNESIA

"THE PERFECT ANTACID"

SUPERIOR TO LIME WATER, BICARBONATE OF SODA, CHALK, ETC.
for correcting Hyperacid conditions—local or systemic.
Vehicle for Salicylates, Iodids, Balsams, etc.

TRADE MARK

PHILLIPS' REFINED CAMPHOR
PHILLIPS' WHEAT PHOSPHATES
PHILLIPS' DIGESTIBLE COCOA
PHILLIPS' PHOSPHO-MURIATE OF QUININE
PHILLIPS' SYRUP OF WHEAT PHOSPHATES
PHILLIPS' EMULSION OF COD LIVER OIL

### A REAL RIP VAN WINKLE

The Strange Case of Dr. Bruno. By F. E. Daniel, M. D. Sold by Von Boeckmann-Jones Company, Austin, Texas. Price, $1.50, or $2.00 with the "Red Back" one year.

Dr. Daniel, the well known editor of the Texas "Red Back" (the *Texas Medical Journal*), has had a quarter of a century of experience as a medical writer, and now, with amazing versatility, he gives us an intensely interesting work of fiction teeming with heart interest. He has blended with the hand of a master, philosophy, religious faith, scientific knowledge, love and romance into an imaginative tale which enthralls the reader from the first page to the close. We were annoyed when compelled to lay it down during our reading, and impatient till we got back to it. The possibility of a synthetic drug inducing prolonged suspended animation is the central theme around which he weaves a weird romance. Well known scientific facts are so deftly threaded among physical impossibilities that the reader must be on his guard lest he dream that astounding discoveries are actually being exemplified and analyzed as demonstrable truths. One feels that the mantle of Jules Verne or Rider Haggard has fallen on worthy shoulders. The doctor who starts to read this book will neglect some of his patients. We know it has thrilled us in a way we have never been thrilled since we first read Ben Hur. The owner will always have a book that he may hand to a friend in assurance that it will be thoroughly enjoyed; but if it is left on the waiting-room table it will surely be stolen.—Dr. A. L. Russell, in *Medical World,* Philadelphia.
